# Trends in clinical trial registration in sub-Saharan Africa between 2010 and 2020: a cross-sectional review of three clinical trial registries

**DOI:** 10.1186/s13063-021-05423-1

**Published:** 2021-07-21

**Authors:** Bassey Edem, Chukwuemeka Onwuchekwa, Oghenebrume Wariri, Esin Nkereuwem, Oluwatosin O. Nkereuwem, Victor Williams

**Affiliations:** 1 Department of Vaccines and Immunity, Medical Research Council (MRC) Unit The Gambia at the London School of Hygiene and Tropical Medicine, Fajara, The Gambia; 2 Unit of Epidemiology and Biostatistics, School of Public Health, University of the Witwatersrand, Johannesburg, South Africa

**Keywords:** Clinical trial registration, Sub-Saharan Africa

## Abstract

**Objective:**

Prospective registration of clinical trials is an ethical, scientific, and legal requirement that serves several functions, including minimising research wastage and publication bias. Sub-Saharan Africa (SSA) is increasingly hosting clinical trials over the past few years, and there is limited literature on trends in clinical trial registration and reporting in SSA. Therefore, we set out to determine the trends in clinical trials registered in SSA countries between 2010 and July 2020.

**Methods:**

A cross-sectional study design was used to describe the type of clinical trials that are conducted in SSA from 1 January 2010 to 31 July 2020. The registries searched were ClinicalTrials.gov (CTG), the Pan African Clinical Trials Register (PACTR), and the International Standard Randomized Controlled Trial Number (ISRCTN). Data were extracted into Excel and imported into STATA for descriptive analysis.

**Results:**

CTG had the highest number of registered trials at 2622, followed by PACTR with 1501 and ISRCTN with 507 trials. Trials were observed to increase gradually from 2010 and peaked at 2018–2019. Randomised trials were the commonest type, accounting for at least 80% across the three registries. Phase three trials investigating drugs targeted at infections/infestations were the majority. Few completed trials had their results posted: 58% in ISRCTN and 16.5% in CTG, thus suggesting reporting bias.

**Conclusion:**

Despite the gradual increase in clinical trials registered during the period, recent trends suggest a drop in the number of trials registered across the region. Strengthening national and regional regulatory capacity will improve clinical trial registration and minimise reporting bias in completed clinical trials.

**Supplementary Information:**

The online version contains supplementary material available at 10.1186/s13063-021-05423-1.

## Background

Clinical trial registration involves prospectively registering a clinical trial’s details in a publicly accessible, web-based database called a clinical trial registry [1]. Historically, clinical trial registration was mandated by the World Medical Association in its 1967 Declaration of Helsinki [2]. The Declaration had, among other things, called for the prospective registration of clinical trials in a publicly accessible platform. It equally tasked collaborators such as investigators, sponsors, and others to have a mechanism to disseminate the results of clinical trials ethically, irrespective of the results. Clinical trial registration is both an ethical and legal obligation in the conduct of clinical trials [3]. The registration of clinical trials serves many purposes, such as providing a publicly accessible repository of trials that may help patients and the public decide which to enrol in, avoid needless repetition of trials, and minimise publication bias by forestalling the selective reporting of research outcomes [4, 5]. Clinical trial data submitted for registration must contain a minimum of twenty-four essential items for the Trial Registration Data Sets (TRDS) to be considered fully registered [6].

Two notable events have positively impacted clinical trial registration. In 2005, the International Committee of Medical Journal Editors (ICMJE) made the prospective registration of clinical trials a requirement for accepting manuscripts reporting study results for publication [7]. Also, in 2006, the World Health Organization (WHO) established the International Clinical Trials Registry Platform (ICTRP) to provide a single point for scientists, patients, and the public to access global clinical trials. In the WHO registry network, registries are classified as primary and partner registries [8]. The primary registries comply with the WHO- and ICMJE-specific criteria for content, quality and validity, accessibility, unique identification, technical capacity, and administration. In addition to complying with some of these requirements, partner registries are not required to have a regional or national mandate, may or may not be managed by a not-for-profit organisation, and accept prospective registration. Currently, there are 17 primary registries in the WHO’s ICTRP network [8].

Although studies have demonstrated an increasing number of clinical trials conducted in Africa, as evidenced by the rising number of trials registered in the continent [6, 7], it is still far less than the rest of the world. Recent estimates in a study that utilised the ICTRP database found that Africa recorded the least registered interventional clinical trials (2.3%) in the WHO ICTRP database from 2004 to 2013, compared to North America, Europe, and Asia that was 35.2, 35.2, and 25.0%, respectively. Others were Oceania at 6% and Latin America and the Caribbean with 4.3% [9].

Sub-Saharan Africa (SSA), a region comprising 48 countries and a population of 1.107 billion people as of 2019 [10], accounts for a large proportion of the global burden of infectious and non-communicable diseases (NCDs) [11]. Over the past decade, the region has been the site of several groundbreaking research into interventions for preventing and treating infectious diseases such as pneumonia, and malaria, [12, 13]. These interventions have not only improved disease control but have transformed global health. There is, however, limited evidence on the types of clinical trials registered in the region compared to other world regions. Our objective, therefore, was to describe the trends in clinical trial registration in the SSA region over the past decade by reviewing three registries where trials conducted on the continent are commonly registered.

## Methods

### Data sources

This study utilised data obtained from ClinicalTrials.gov (CTG), the Pan African Clinical Trial Registry (PACTR), and the International Standard Randomized Controlled Trial Number (ISRCTN) registries. Established in 2000, CTG (https://www.ClinicalTrials.gov/ct2/about-site/background) is a collaborative effort of the US National Institute of Health (NIH) and the Food and Drug Administration (FDA). It serves as a resource to provide accessible information on publicly and privately funded research involving human subjects [14]. The PACTR, on the other hand, is a regional registry of clinical trials conducted within Africa. It was established in 2007 as the AIDS, TB, and malaria clinical trial registry, and by 2009, its mandate had broadened to include clinical trials in all fields [15]. Lastly, the ISRCTN was launched in 2000 and serves as a primary registry for all types of intervention studies involving human subjects [16]. We performed a preliminary analysis of the source registry for study records conducted in SSA, available in the WHO’s ICTRP, and found that the CTG, PACTR, and ISRCTN together represent about 88% of ICTRP data source (analysis not shown), hence, our decision to utilise records in these three registries. All three registries are part of the WHO ICTRP and are publicly accessible, with both PACTR and ISRCTN designated as primary registries [8].

### Search and inclusion

**Fig. 1:**
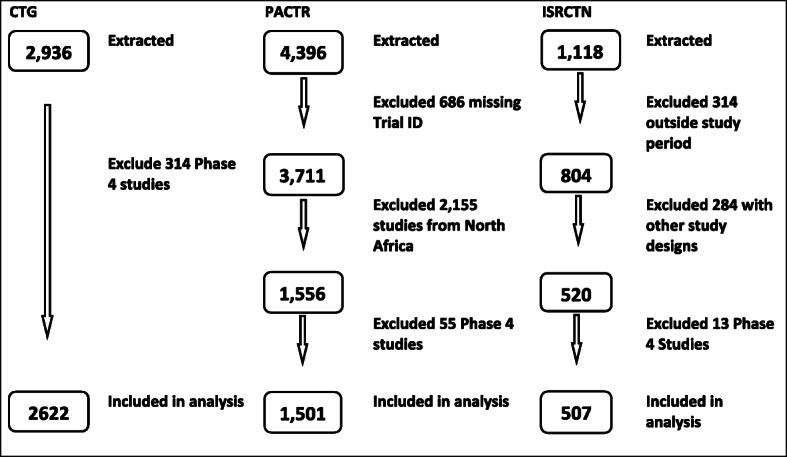
Flow chart describing data extraction by registry. The three registries had varying levels of flexibility during data extraction. With CTG, it was possible to apply filters directly for countries in SSA for the study period. PACTR and ISRCTN had limited flexibility, and components of the data which was not required are excluded as shown in the flow chart

We conducted a basic search of the CTG, PACTR, and ISRCTN on 15 August 2020, 18 August 2020, and 9 September 2020, respectively. We searched by country for each registry and identified all clinical trials on human subjects registered between 1 January 2010 and 31 July 2020. We decided to include the first half of 2020 in our analysis to explore how the clinical trial landscape has fared during the COVID-19 pandemic. Trials were eligible if they included participants from an SSA country. Only interventional studies were eligible to be included in this research. We excluded observational, post-licencing, and impact evaluation studies. For all three registries, we re-categorised the data as presented. The flowchart in Fig. 1 describes our search results and which studies were eligible across all three registries.

### Data management

We downloaded the records from the registries in separate Excel files for each eligible country. The registries had a maximum number of records that could be extracted at a time. To overcome this challenge, records were extracted monthly, quarterly, or annually depending on the number of records until all the study period records were extracted. The extracted country records from each registry were subsequently cleaned and merged into a single dataset for analysis. We extracted data on unique trial identifiers, trial registration history, the status of the trial, participant description, trial administration, publication status, and study sponsors for each registered trial. Details of complete data items extracted are presented in Table 1.

**Table 1:** Data items extracted for all trials

Primary registry ID	
Study title	
Countries of recruitment	
Date of trial registration	
Timing of registration (relative to study start date)	
Study start date	
Recruitment start date	
Expected date of study completion (or end)	
Study status	
Study phase	
Primary study design	
Health condition addressed by the trial	
Type of intervention studied	
Participant age group	
Participant gender	
Type of participant studied^a^	
Publication of results	
Publication of trial protocol	
Study sponsor	

^a^Available only in ISRCTN

In all three registries, study sponsors were entered as free text. Thus, we identified the various study sponsors by extracting and grouping the common recurring themes such as individuals’ titles, university/faculty/school/academic institutions, hospital/clinic/medical centre, or research institute/organisation in the descriptive text. We performed a similar data management process for study design and the age of study participants in CTG. All three registries had varying completeness levels as specific variables which were either absent or had > 90% missing depending on the registry were excluded from the analysis. Individual observations missing unique identification numbers were dropped, and data items were described relative to the total number of trials with unique study identification for the study period. We performed data management with Excel and STATA 15 (STATA Corp., College Station, TX).

### Analysis

A descriptive analysis using tables and figures was used to examine the trends in clinical trial registration in SSA for the period under review. Summary statistics of specific items were described for the three registries using proportions (Table 2). We presented a summary of completed trials with results for all countries in SSA in the three registries in a table (Appendix I). We also illustrated the total number of trials registered by year in each registry in a line graph. The top ten countries with the highest number of entries for each registry and the trials’ results status were also presented. A sub-analysis of data from CTG to identify the commonest intervention type registered and the primary purpose of the study was done (Appendix II).

**Table 2:** Summary characteristics of trials in the three registries

Description	ISRCTN, ***N*** (%)	CTG, ***N*** (%)	PACTR, ***N*** (%)
**Number of trials registered**	507	2622	1501
**Number of studies completed**	386 (76.1)	1469 (56.0)	-
**Number of completed with results**	224 (44.2)	243 (9.3)	40 (2.6)
**Overall trial status**			
Completed	386 (76.1)	1469 (56.0)	-
Ongoing/active	103 (20.3)	278 (10.6)	-
Not yet recruiting/pending	15 (3.0)	147 (5.6)	443 (29.5)
Recruiting	49 (9.7)	429 (16.4)	324 (21.6)
Stopped/terminated	2 (0.4)	65 (2.5)	-
Suspended	16 (3.2)	18 (0.7)	-
Withdrawn	-	34 (1.3)	-
Other/unknown	-	183 (7.0)	-
**Prospective/retrospective**			
Prospectively registered	228 (45.0)	-	-
Retrospectively registered	279 (55.0)	-	-
**Study design**			
**Allocation**			
Randomised	450 (88.8)	2090 (79.7)	1319 (87.9)
Non-randomised	50 (9.9)	224 (8.5)	175 (11.7)
N/A	3 (0.6)	300 (11.4)	7 (0.5)
**Intervention model**			
Parallel assignment	-	1919 (73.2)	1003 (66.8)
Single group assignment	-	414 (15.8)	49 (3.3)
Cross-over assignment	-	113 (4.3)	95 (6.3)
Factorial assignment	-	107 (4.1)	341 (22.9)
Sequential assignment	-	60 (2.3)	-
None (open label)	-	7 (0.3)	-
**Masking**			
None	-	1577 (60.1)	-
Single	-	348 (13.3)	-
Double	-	224 (8.5)	-
Triple	-	146 (5.6)	-
Quadruple	-	312 (11.9)	-
**Phase**			
Not applicable	149 (29.4)	1426 (54.4)	1301 (86.7)
Not specified	10 (2.0)		
Phase 0/early phase 1	-	11 (0.4)	43 (2.9)
Phase I	4 (0.8)	169 (6.4)	43 (2.9)
Phase I/II	3 (0.6)	109 (4.2)	
Phase II	27 (5.3)	371 (14.1)	52 (3.5)
Phase II/III	10 (2.0)	113 (4.3)	
Phase III	57 (11.2)	423 (16.1)	60 (4.0)
Phase III/IV	2 (0.4)		
**Sponsor**			
University	262 (51.7)	1618 (61.7)	496 (33.3)
Industry/INGO^a^	16 (3.2)	288 (11.0)	27 (1.8)
Research institution	105 (20.7)	1190 (45.4)	164 (10.9)
Government	56 (11.0)		
Charity	38 (7.5)		
Hospital/clinic	16 (3.2)	437 (16.7)	94 (6.3)
Private individuals			116 (7.7)
**Sex**			
Both	413 (81.5)	1961 (74.8)	1074 (71.6)
Female	88 (17.4)	553 (21.1)	360 (24.0)
Male	5 (1.0)	108 (4.1)	67 (4.5)
**Target number of participants**			
Min, max^a^	0, 2,000,000	0, 650,000	0, 497,379
Mean (SD)^a^	73,780 (338,098)	5298 (32,513)	1322 (15,010)
Median (IQR)^a^	902 (2,004,000)	348 (1,001,233)	140 (60,400)

*INGO* international non-governmental organisations

^a^This will not equal total completed trials because sponsor type was obtained by extracting the common theme from a free text, indicating the possibility of a double count

## Results

### Summary of search results

Table 2 summarises the characteristics of different items from each of the registries under review. Different registries reported standardised characteristics using different approaches with a varying level of completeness for each item. CTG had the highest number of registered trials at 2622, followed by PACTR at 1501 and ISRCTN with 507 trials. Only the ISRCTN contained information on the timing of trial registration, that is, whether a trial is prospectively or retrospectively registered. Of the 507 clinical trials reported, 45% were prospectively registered, while 55% were registered retrospectively. ISRCTN had the highest proportion of completed trials, and greater than 80% of trials in the three registries were randomised. The parallel assignment was the commonest intervention model as shown by CTG and PACTR, which documented this information with cross-over, single group, and factorial featuring prominently.

Masking was not done in about 60% of trials as reported by CTG only, and where present, single (13.3%) and quadruple (12%) masking were the preferred methods. Data on the trials’ phase were complete in CTG and PACTR (100%), while ISRCTN had missing data in 245 (48%) of 507 included trials. Over 50% of reported trials had their clinical trial phase classified as “not applicable” or “not specified” from the available data. In PACTR, this proportion was higher at 86%, and trials in early phase 1 to phase III ranged from 0.42 to 16.1%.

Only the ISRCTN reported on disease conditions that the trials targeted (Appendix III). Most of the registered trials involved interventions addressing infectious diseases and infestations (42.5%). A further 12% concerned maternal and newborn conditions and 6.5% were related to mental and behavioural conditions. No specific disease condition was mentioned in 55 of the identified trials. Top sponsors from the three registries were universities or academic institutions, closely followed by research organisations. The three registries did not have variables describing the countries sponsors of the study were based to enable analysis. However, a review of the ISRCTN Registry (with few entries) shows that 6–50% of studies had sponsors from the countries the studies were conducted in the top ten countries. This is presented in Table 3. This process was not possible for CTG, but a review of the registry, however, shows that most of the sponsors are US-based with a country-based sponsor or collaborator. Similarly, this analysis was not feasible with PACTR, but the authors observed the portal is presently being updated and may enable this analysis in future.

**Table 3:** Summary of top ten countries with same country sponsor

Country	Number of studies	Same country sponsor	% Same country sponsor
Ghana	22	11	50%
South Africa	66	32	48%
Nigeria	36	16	44%
Zambia	24	9	38%
Kenya	47	15	32%
Uganda	82	18	22%
Cote d’Ivoire	14	3	21%
Malawi	43	5	12%
Tanzania	41	5	12%
Ethiopia	18	1	6%

Most studies targeted adults of both sexes (> 70%). Still, limitations with the classification of the age of participants across the three registries made it impossible to properly categorise participants’ age. Only ISRCTN provided information describing the type of study participants, and the majority were patients at 66% (Appendix IV).

### Top countries registering trials

**Fig. 2:**
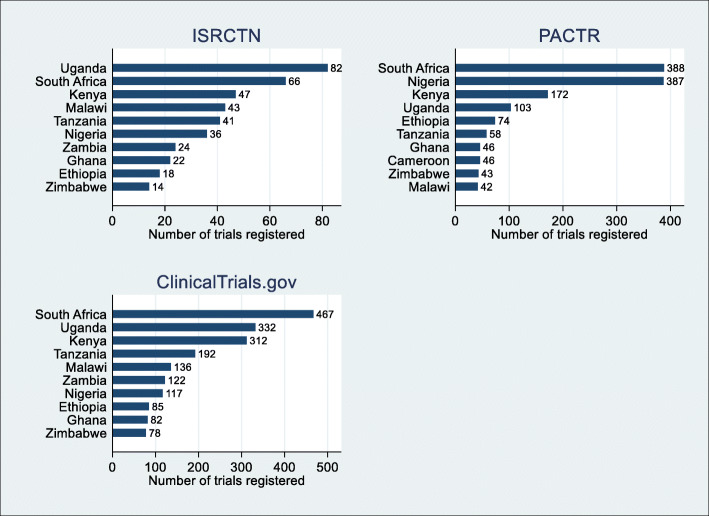
Top ten countries where the highest numbers of trials are registered in sub-Saharan Africa between 2010 and 2020

A review of the three registries indicated that certain countries had more trials registered than other countries in the region. Figure 2 below shows the top ten countries with the highest number of registered trials by each registry. South Africa, Uganda, Kenya, and Nigeria accounted for most of the registered trials, while other major trial sites include Malawi, Tanzania, Zambia, Ghana, Ethiopia, and Cameroon. ClinicalTrial.gov and PACTR were the preferred registries.

### The trend in trial registration

**Fig. 3:**
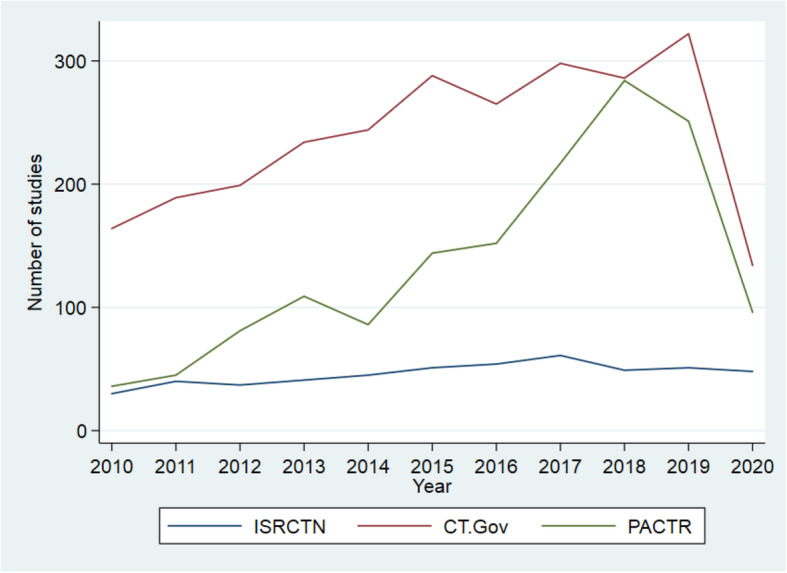
Trends in clinical trial registration in sub-Saharan Africa between 2010 and 2019 across three registries

Between 2010 and 2019, we observed a steady increase in the number of trials registered in CTG, with 322 trials registered in 2019 compared with 164 in 2010. Similarly, the number of trials registered in PACTR increased between 2010 (*n* = 36) and 2018 (*n* = 284), with a decline to 251 in 2019 (data as of 31 July 2020). The number of registered trials on ISRCTN was lowest in 2010 (*n* = 30), peaking in 2017 (*n* = 61), and with a slight dip in 2018 and 2019. Between January and July of 2020, the number of trials registered on ISRCTN is similar to the total number registered in the preceding year. Meanwhile, the numbers registered in the same period in PACTR (*n* = 108) and CTG (*n* = 134) were 40 and 42% of the numbers reported in the preceding year, respectively (Fig. 3).

### Intervention type and purpose of the study

Data on intervention type and the primary purpose of studies was available only in ClinicalTrials.gov (Appendix II). The commonest intervention type was drug (31.9%) closely followed by behavioural interventions at 20%. Other prominent types of intervention documented were biological (11%), device (6.7%), and dietary supplement (5.7%). Treatment accounted for the highest primary purpose of conducted trials at 39.2%. This was closely followed by prevention (33.3%), and health services research (9.4%). Less significant reasons for conducting trials were screening (1.1%) and device feasibility (0.2%).

### Publication of trial results

**Fig. 4:**
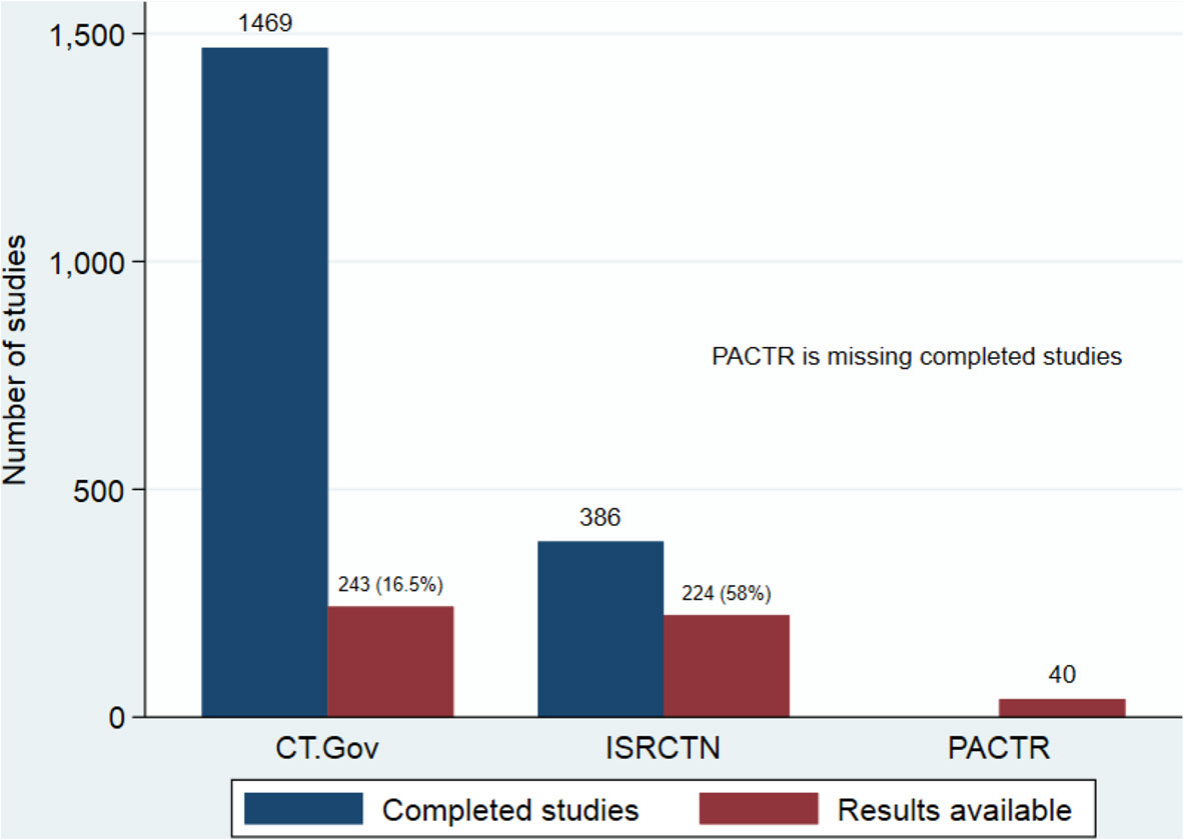
Completed clinical trials with results by registry

Of the 1469 completed trials registered on CTG, only 243 (16.5%) were reported as having results, while 224 of the 386 completed trials registered on ISRCTN had posted a summary of the results (58.0%) (Fig. 4). PACTR registered 40 trials with results but did not provide information on the total number of completed trials. A breakdown of completed trials with results by the country for each registry was analysed and presented as Appendix I.

We further explored the data to identify the proportion of completed trials with published results based on clinical trials’ sponsors across the two registries with complete data (Table 4). Overall, CTG, which had more completed trials, had a lower proportion of completed trials with available results than ISRCTN. The proportion of completed trials with results ranged from 8.1 to 21.2% in CTG depending on the sponsor, while in ISRCTN, it was 50% and slightly above depending on the sponsor except for industry which was 40%.

**Table 4:** Summary of sponsors with published results

Sponsor category	ISRCTN (***N*** = 507)				CTG (***N*** = 2262)			
	Registered (*N*)	Complete (*n*)	Published (*n*)	Proportion of completed with results (%)	Registered (*N*)	Complete (*n*)	Published (*n*)	Proportion of completed with results (%)
University	262	183	108	59.0	1618	891	72	8.1
Industry/INGO^a^	16	15	6	40.0	288	165	30	18.2
Research institutions	105	81	47	58.0	1190	605	104	17.2
Government	56	51	28	54.9	-	-	-	-
Charity	38	31	18	58.1	-	-	-	-
Hospital/clinic	16	12	6	50.0	437	231	49	21.2

^a^*INGO* international non-governmental organisations; this will not equal total completed trials because sponsor type was obtained by extracting the common theme from a free text, indicating the possibility of a double count

## Discussion

Since 2010, there has been some progress in the number of clinical trials registered in SSA. While we cannot account for the decline in clinical trial registration observed in ISRCTN and CTG from 2018 to 2019, a possible explanation for this may be that more trials are registered in other registries as the number of available registries at investigators’ disposal increases. The consistency in the number of registered trials observed in the ISRCTN is suggestive of this. We did not observe a large difference in the number of registered clinical trials in the first half of 2020 compared to the preceding year, despite the increase in COVID-19 research. While much of the current focus of biomedical research has been on COVID-19 interventions, we identified only one clinical trial of a COVID-19 candidate vaccine registered in Africa. However, we believe this would improve as plans are underway to ensure Africa hosts more clinical trials of COVID-19 interventions. In fact, not only has a second COVID-19 vaccine trial commenced in Kenya but a consortium of African researchers and research institutions have been launched to facilitate the conduct of COVID-19-related trials on the continent [17]. Also, considering that historically, the proportion of clinical trials registered in low- and middle-income countries (LMICs) lag behind high-income countries (HIC), and most SSA countries are LMICs. Despite our analysis being restricted to this region alone, our findings suggest the same trend compared to existing literature [9].

From our study, infections and infestations were the top health condition researched. Despite this data being available from only one registry (ISRCTN), it is a useful pointer that the trials conducted align with the region’s health needs. A review of the PACTR performed 6 years after its launch revealed that infectious diseases were the leading condition researched on the African continent [18]. In contrast, in Egypt, which is not in sub-Saharan Africa, cancers were the most studied conditions [19]. Recent evidence suggests an epidemiological transition with NCDs becoming the leading source of morbidity and mortality on the continent [11], indicating a need to prioritise research into these conditions.

Study sponsors are mandated to summarise their study findings in the registry and disseminate them in peer-reviewed publications to improve transparency [20, 21]. Our study reveals that most of the clinical trials conducted in SSA go unreported, considering that CTG, which had the highest number of completed trials, had less than 20% of the trials reported. In contrast, more than half of those in the ISRCTN were reported. While there is limited literature on this important trend in SSA generally, a study found that about 48.6% of completed trials in the South African National Clinical Trials Register (SANCTR) were registered within a median time of 2 years of completion and the majority of the published trials were those with a positive outcome [22], revealing the possibility of publication bias. Our study relied solely on completed trials with results available in the study field and did not further verify if these studies have been published in peer-reviewed journals as was done in the South African study. Despite this difference in the methodology, we demonstrate that publication bias is a problem that needs to be addressed. However, study sponsors’ failure to register trials and post summary outcomes of completed studies persists even with stringent regulatory authorities in developed countries [23, 24]. A study designed differently from ours found that about 15% of 439 trials were registered retrospectively, after the investigators may have observed some of the primary outcomes [25]. It is, however, noteworthy that investigators may opt to publish study results in journals rather than post them in the clinical trial registry or do either of these at different time intervals, as has been established in the literature [26, 27].

Research is unevenly distributed across the continent, with Uganda and South Africa accounting for over 50% of the registered studies. These countries have well-established biomedical research capacity, which could explain the higher clinical trial output from these countries. Indeed, these countries host many of the major biomedical research institutions in sub-Saharan Africa, according to the SCIimago institute ranking [28]. Due to their reputation, these high-ranking institutions are more likely to attract clinical trials and other types of research.

Our findings should, however, be interpreted with caution. Firstly, we analysed the data available in only three registries. Despite this limitation, we believe that our results give a realistic picture of clinical trial registration trends for the past decade in SSA. Our analysis of the data sources for the available trials in the WHO’s ICTRP found that CTG, PACTR, and ISRCTN together represent about 88% ICTRP data source (analysis not shown). They, therefore, provide an adequate representation of clinical trials registered from sub-Saharan Africa. Next, we did not de-duplicate study records found in all three registries as each registry assigned a unique study identification number (id) to each trial. Consequently, individual study records could be found in one or more registries but with a different study id. Hence, analysing the records gives the unique advantage of presenting a snapshot of trial registration in SSA in any registry. Missing data across the submitted trials in the three registries may also have the potential to bias some of our findings. These range from fields that were completed as “unknown” or missing to others, such as the absence of trial status in the PACTR. For the latter, we were unable to estimate with certainty the proportion of completed trials in that registry that had results posted. The problem of incomplete trial datasets in registries has been highlighted [29, 30] and still remains a challenge across registries.

Also, despite existing regulations on prospective registration of clinical trials, some study sponsors fail to comply or only register their trials retrospectively [25, 31]. This impacts our study because we do not have a useful denominator for all the trials that have been conducted in Africa. Nevertheless, the records of the registries are useful and provide vital information required to improve the documentation of clinical trials in Africa. One outcome of our study, the proportion of completed clinical trials with results available, may be impacted by the “lag” time between completion of a trial and posting of results.

The various registries’ quality control teams need to pay greater attention to the submitted entries and ensure complete, comparable, and verifiable records. This is vital as we noted a great variability in the information documented in the three registries. Ensuring similar data fields in registries will not only be beneficial to the public but will enable ease of comparability and analysis of such data that can inform policy formulation. Finally, we recommend assigning each clinical trial a unique code for registration across all clinical trial registries to enhance the de-duplication of study data across and within registries during analysis of such data.

A clinical trial authorisation is one of the core functions of National Regulatory Authorities (NRAs) and these agencies, although available in all but one African country, have limited capacity [32, 33]. However, several initiatives are in place to strengthen regulatory capacity across the African continent. Some of these are regional regulatory harmonisation initiatives, such as the African Medicines Regulatory Harmonization (AMRH) and African Vaccine Regulatory Forum (AVAREF) [34], and the North-South collaborative initiatives like the European and Developing Countries Clinical Trials Partnership (EDCTP). These initiatives would possibly impact not just the conduct of trials in SSA but also the prospective registration and subsequent posting of results of completed trials that is an international best practice.

## Conclusion

Despite the highlighted limitations of our study, we have demonstrated an uneven but sustained rise in the number of clinical trials registered in SSA using the records in three clinical trial registries. This was, however, followed by a sharp decline between 2018 and 2019. Phase three trials investigating drugs targeted at infections/infestations were the majority. In over half of the completed trials, study sponsors failed to summarise the trial outcomes as mandated by regulatory guidelines. Although regulatory capacity is limited across the continent, several ongoing initiatives aim to improve ethics and regulatory capacity in Africa. This, hopefully, will lead to an improvement in clinical trial conduct, registration, and publication of study results.

## Supplementary Information

**Additional file 1: Appendix I: Table 1.** Summary of completed studies with results for all the countries. **Appendix II**. Intervention type and the primary purpose of study. **Table 2.** Intervention Type (CTG. ONLY). **Table 3.** Primary purpose of study (CTG ONLY). **Appendix III: Figure 1.** Disease type targeted by trials. **Appendix IV: Figure 2.** Type of Study Participants

## Data Availability

The datasets used and/or analysed during the current study are available from the corresponding author on reasonable request.

## Abbreviations

CTG: ClinicalTrials.gov
ICTRP: International Clinical Trials Registry Platform
ISRCTN: International Standard Randomized Controlled Trial Number
LMICs: Low- and middle-income countries
NCDs: Non-communicable diseases
PACTR: Pan African Clinical Trials Register
SSA: Sub-Saharan Africa
WHO: World Health Organization
