# Supplementary material for: Trends in clinical trial registration in sub-Saharan Africa between 2010 and 2020: a cross-sectional review of three clinical trial registries
Source: Trials. 2021 Jul 21;22:472. doi: 10.1186/s13063-021-05423-1 (PMC8293494; doi:10.1186/s13063-021-05423-1)

**Supplementary material**

**Table of Contents**

[Appendix I 2](#_Toc62048467)

[Appendix II: Intervention type and primary purpose of study 4](#_Toc62048468)

[Appendix III 5](#_Toc62048469)

[Appendix IV 6](#_Toc62048470)

# Appendix I

**Table 1: Summary of completed studies with results for all the countries**

| **Country** | **ISRCTN** | | **CT.Gov** | | **PACTR** | |
| --- | --- | --- | --- | --- | --- | --- |
|  | **Completed  studies** | **Results  available** | **Completed  studies** | **Results  available** | **Completed  studies** | **Results  available** |
| Angola | 3 | 0 | - | - | - | - |
| Benin | 5 | 3 | 12 | 2 | - | - |
| Botswana | - | - | 15 | 5 | - | - |
| Burkina Faso | 10 | 6 | 47 | 12 | 6 |  |
| Burundi | 1 | 0 | 9 | 0 | - | - |
| Cameroon | 7 | 5 | 21 | 2 | 7 |  |
| Central African Republic | - | - | 2 | 0 | - | - |
| Chad | - | - | 3 | 0 | - | - |
| Congo | - | - | 18 | 3 | - | - |
| Cote d'Ivoire | 12 | 9 | 14 | 0 | 1 |  |
| DR Congo | 6 | 3 | 20 | 1 | 3 |  |
| Equatorial Guinea | - | - | 3 | 0 | - | - |
| Eritrea | - | - | 1 | 0 | - | - |
| Eswatini | - | - | 8 | 0 | - | - |
| Ethiopia | 14 | 7 | 52 | 4 | 18 | 6 |
| Gabon | - | - | 10 | 1 | - | - |
| Gambia | 11 | 7 | 23 | 5 | 4 |  |
| Ghana | 17 | 8 | 46 | 7 | 8 | 2 |
| Guinea | 3 | 2 | 6 | 2 | 2 | 0 |
| Guinea Bissau | 2 | 0 | 2 | 1 | - | - |
| Kenya | 40 | 19 | 193 | 31 | 73 | 10 |
| Lesotho | 1 | 0 | 5 | 0 | - | - |
| Liberia | - | - | 6 | 1 | - | - |
| Madagascar | 2 | 1 | 4 | 0 |  |  |
| Malawi | 36 | 25 | 71 | 22 | 14 | 2 |
| Mali | 6 | 2 | 36 | 3 | 5 | 0 |
| Mauritius | 1 | 1 | 14 | 3 | - | - |
| Mozambique | 3 | 2 | 21 | 3 | - | - |
| Namibia | - | - | 1 | 0 | - | - |
| Niger | 3 | 1 | 6 | 0 | 1 |  |
| Nigeria | 31 | 17 | 61 | 9 | 155 | 10 |
| Rwanda | 3 | 0 | 41 | 5 | 4 | 0 |
| Senegal | 2 | 2 | 28 | 6 | 10 | 0 |
| Sierra Leone | 4 | 1 | 17 | 3 | 1 | 0 |
| Somalia | 2 | 1 | 1 | 0 | - | - |
| South Africa | 50 | 27 | 279 | 64 | 125 | 9 |
| South Sudan | 1 | 1 | - | - | - | - |
| Sudan | 7 | 1 | 9 | 0 | 3 | 0 |
| Tanzania | 30 | 21 | 100 | 12 | 18 | 0 |
| Togo | 2 | 1 | 2 | 1 | - | - |
| Tunisia | - | - | - | - | 12 | 0 |
| Uganda | 46 | 34 | 154 | 20 | 49 | 1 |
| Zambia | 18 | 12 | 72 | 6 | 6 | 0 |
| Zimbabwe | 7 | 5 | 35 | 9 | 14 | 0 |

# Appendix II: Intervention type and the primary purpose of study

**Table 2: Intervention Type (CT.GOV ONLY)**

| **Intervention type** | **Frequency** | **Percent** |
| --- | --- | --- |
| Drug | 836 | 31.88 |
| Behavioral | 524 | 19.98 |
| Other | 471 | 17.96 |
| Biological | 290 | 11.06 |
| Device | 175 | 6.67 |
| Dietary Supplement | 149 | 5.68 |
| Procedure | 89 | 3.39 |
| Diagnostic Test | 57 | 2.17 |
| Combination Product | 28 | 1.07 |
| Radiation | 3 | 0.11 |

**Table 3: Primary purpose of study (CT.GOV ONLY)**

| **Primary purpose of study** | **Frequency** | **Percent** |
| --- | --- | --- |
| Treatment | 1002 | 0.392 |
| Prevention | 852 | 0.333 |
| Health Services Research | 242 | 0.094 |
| Diagnostic | 141 | 0.055 |
| Other | 127 | 0.05 |
| Supportive Care | 108 | 0.042 |
| Basic Science | 51 | 0.02 |
| Screening | 28 | 0.011 |
| Device Feasibility | 6 | 0.002 |

# Appendix III

**Figure 1: Disease type targeted by trials**


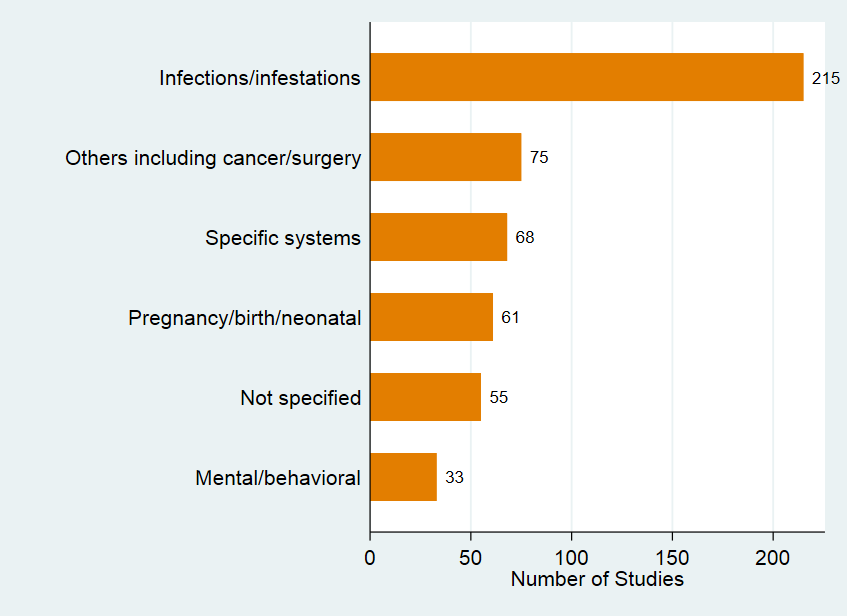


# Appendix IV

**Figure 2: Type of Study Participants**


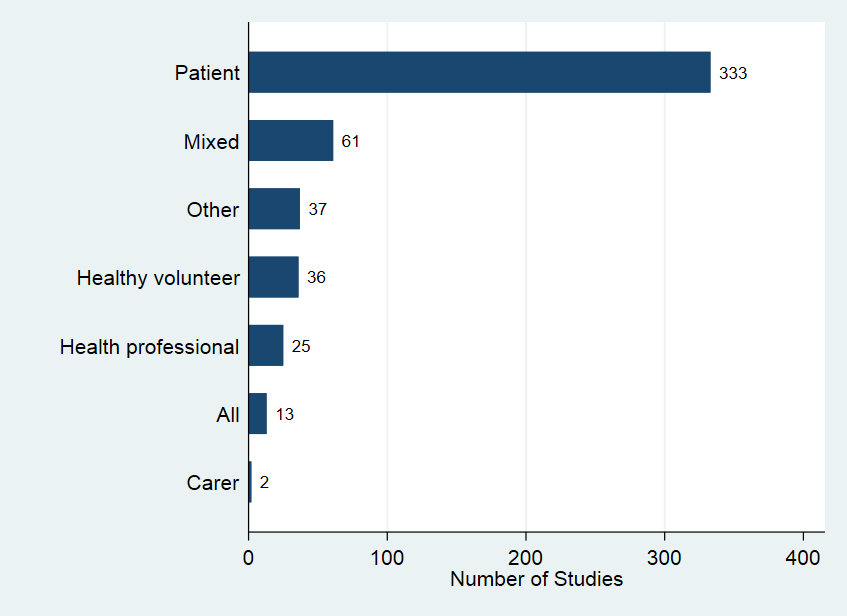

Supplement: Supplementary file 1 — Additional file 1: Appendix I: Table 1. Summary of completed studies with results for all the countries. Appendix II. Intervention type and the primary purpose of study. Table 2. Intervention Type (CTG. ONLY). Table 3. Primary purpose of study (CTG ONLY). Appendix III: Figure 1. Disease type targeted by trials. Appendix IV: Figure 2. Type of Study Participants [file 13063_2021_5423_MOESM1_ESM.docx]
